# 

**Published:** 1887-01-01

**Authors:** 


### TABLE OF CONTENTS.

### Original and Selected Articles.

A Caution Against Septic Germicides, by J. McF Gaston, M. D. .... 1
How to Avoid the Taste of Medicine, by A. F. Durham, M.D., of Georgia .... 4
Observations at the Bedside in a Number of Cases and Diseases, by S. B. Bouchelle, M. D., of Thomasville, Ga. .... 5
Rare Effects of Quinine on the Skin, by C. H. Wilkinson, M. D. .... 8
A Case of Pseudo-Epilepsy of the Retina Caused by Hypermetropia, by F. C. Riley, M.D., of New York .... 11
On Some New Hypnotics, by Dr. S. G. Webber... 13
The Prognosis of Croup .... 44
Resection of the Entire Shaft of the Tibia for Necrosis, with Reproduction of the Bone—Recovery .... 15

### Abstracts and Gleanings.

Recent Progress in the Investigation of Hog Cholera .... 17
Poisoning by Milk .... 18
Investigations on the Action of Micrococci of Vaccine .... 19
Note on the Treatment of Thread-worms in Children; Vertigo .... 20
Stone in the Bladder—Its Surgical Treatment; Typhoid Fever .... 21
Podophyllum Hypodermically; Laparotomy as an Aid to Herniotomy; She Was Not Spayed .... 22
Treatment of Pruritus Pudendi .... 23
Cholera .... 24
Incontinence of Urine; The Water Diet .... 25
Potassium Permanganate in Amenorrhœa; Antiseptic Treatment of Carbuncle; An Incompatible of Antipyrine; An Item .... 26
Ingluvin .... 27
Surgical Myxœdema; For Gleet and Chronic Gonorrhœa .... 29
A New Hæmostatic; Ophthalmia Neonatrum; Geoslin .... 30
Substitute for Fehling's Solution; Lister's Latest Antiseptic Dressing; Hemirheumatism; Ingrowing Toe-nail .... 31
Calomel in Diphtheria; Hepatic Colic; Albuminura; Barbasco—A New Mexican Drug .... 32

### Scientific Items.

Rare Metals; Aerial Navigation; Another Electric Motor .... 33
Late Discoveries .... 34

### Practical Notes and Formulæ.

Superior Carminative; A Good Styptic; Colic in Children; An Application for Pruritus Vulvæ; Hypodermic Solution of Quinine .... 35

Cutaneous Anodyne; Treatment of Acute and Chronic Nasal Catarrh; Chionanthus Virginica in Jaundice .... 36

### Editorials and Miscellaneous.

Editorial Brevities .... 37
Our City Physicians .... 39
Laws Against Quackery .... 40
Professional Integrity .... 43
Receipted; Special Notes .... 44

